# Prognostic Role of NLR in Urinary Cancers: A Meta-Analysis

**DOI:** 10.1371/journal.pone.0092079

**Published:** 2014-03-18

**Authors:** Yong Wei, Ya-Zhi Jiang, Wen-Hui Qian

**Affiliations:** Department of Urology, Gaochun County Hospital, Nanjing, China; Shanghai Jiao Tong University School of Medicine, China

## Abstract

**Background:**

Recently, many studies explored the role of inflammation parameters such as neutrophil-to-lymphocyte ratio (NLR) in the prognosis of urinary cancers, but the results were not consistent.

**Methods:**

We carried out a meta-analysis of published studies to assess the prognostic value of NLR in patients with urinary cancers. Hazard ratio (OR) with 95% confidence interval (CI) was used to assess the association of NLR and OS and RFS/CSS.

**Results:**

The pooled results showed that high NLR was a poor predictor for OS with HR of 1.81 (95%CI: 1.48–2.21; P_heterogeneity_ = 0.005) and RFS/CSS (HR = 2.07, 95% CI: 1.65–2.6; P_heterogeneity_ = 0.849). Subgroup analyses revealed that high NLR yielded a worse OS in RCC (HR = 1.9, 95%CI: 1.47–2.45; P_heterogeneity_ = 0.003) and a poor RFS/CSS in RCC (HR = 1.83, 95%CI: 1.35–2.48; P_heterogeneity_ = 0.709), bladder cancer (HR = 2.2, 95%CI: 1.27–3.8; P_heterogeneity_ = 0.447) and urothelial carcinoma (HR = 2.58, 95%CI: 1.66–4.01; P_heterogeneity_ = 0.784).

**Conclusion:**

Our results showed that NLR could act as a significant biomarker in the prognosis of urinary cancers.

## Introduction

Due to the aging and growth of population as well as an increasing adoption of cancer-associated lifestyle such as smoking and “westernized” diets, the global burden of cancer continues to increase [1], [2]. Urinary cancers including prostate cancer, renal cell cancer and bladder cancer are common types of malignancies worldwide especially in western countries. Although survival in patients with urinary cancers has improved in recent years due to the advance in treatment modalities such as sipuleucel-T based immunotherapy [3] and application of molecular targeted drugs [4], [5], a subset of patients died within a few months following surgery due to the rapid progression of disease [6]. Lack of efficiently prognostic biomarkers is partly responsible for the high mortality rates caused by cancer. Thus, efficiently and reliable biomarkers for providing additional prognostic information are urgently needed.

As a marker of systemic inflammatory response, neutrophil-to-lymphocyte ratio (NLR) has been studied as a useful prognostic biomarker in various cancers such as lung cancer [7] and colorectal cancer [8]. Elevation of NLR in patients with urinary cancers always predicted a worse prognostic outcome, but some studies [9]–[12] presented inconsistent results such as the study of Shafique et al. and Linton et al.. Therefore, this meta-analysis was conducted to reveal the prognostic value of NLR in urinary cancers. To our knowledge, it is the first meta-analysis to investigate the prognostic role of NLR in urinary cancers.

## Materials and Methods

### Publication search and inclusion criteria

Medical subheading (Mesh) terms relating to NLR (e.g. “neutrophil-to-lymphocyte ratio” or “neutrophil lymphocyte ratio”) combined with words related to urinary cancers (e.g. “renal cancer”, “bladder cancer”, “prostate cancer”, “urothelial cancer” or “transitional cell carcinoma”) and terms to prognosis (e.g. “outcome”, “prognosis”, “prognostic” or “survival”) were searched on PubMed and the last search was updated on November 17, 2013. The references of articles and reviews were also explored to retrieve potentially additional studies. Studies were eligible if they met the following criteria: (a) patients with urinary cancers in the studies were histopathologically confirmed; (b) investigated the association of pre-treatment NLR with overall survival (OS), recurrence-free survival (RFS) or cancer-specific survival (CSS); (c) full text articles in English. Exclusion criteria were as follows: (a) letters, reviews, expert opinions, case reports or laboratory studies; (b) studies had overlapping or duplicate data; (c) lack of key information for further analysis.

### Data extraction

Data from each study were evaluated and extracted independently by two investigators (Wei and Jiang). The quality of studies was assessed according to the Dutch Cochrane Centre proposed by Meta-analysis of Observational Studies in Epidemiology (MOOSE) [13]. The following items were recorded: first author's name, year of publication, country, ethnicity, stage, cancer type, total number of cases, cut-off value, follow ups and HRs with 95% CIs. If not available, data were extracted to calculate HR by the method of Tierney et al. [14]. A consensus was reached on each item among the authors in case of discrepancies.

### Statistical analysis

HRs with their 95% CIs from each study were used to calculate pooled HRs. A test of heterogeneity of pooled results was performed using Cochran's Q test and Higgins I-squared statistic. A P<0.10 for Q-test was considered statistically significant, and the random-effects model (DerSimonian-Laird method) was applied to calculate the pooled HRs [15]. Otherwise, the fixed-effects model (Mantel-Haenszel method) was performed [16]. Publication bias of literatures was evaluated using Begg's funnel plot and the Egger's linear regression test and a p<0.05 was considered significant. Trim and fill method was used to assess potential asymmetry in the funnel plot. All statistical analyses were performed using STATA software version 12.0 (STATA Corporation, College Station, TX, USA). And all *P* values were two-sided.

## Results

### Study characteristics

**Figure 1:**
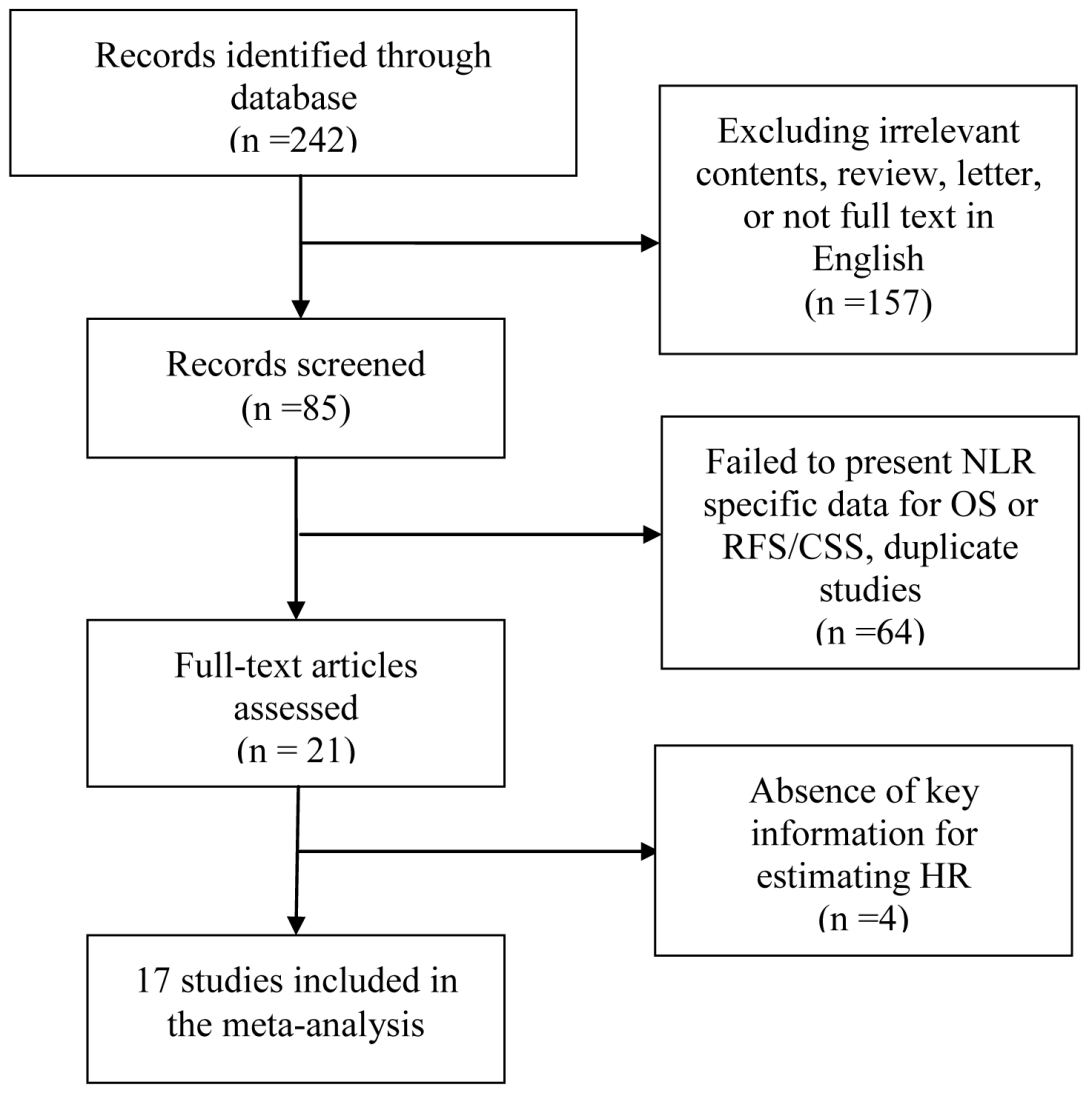
Methodological flow diagram of the meta-analysis.

A total of 17 articles [9]–[12], [17]–[29] were retrieved according to the inclusion and exclusion criteria after careful read and selection. The detailed screening process was shown in Figure 1. Thirteen of 17 articles investigated the prognostic role of NLR for OS, 4 for RFS and 5 for CSS, respectively. Shafique et al. [11] presented separate data before and after diagnosis and Yoshio Ohno et al. [19] investigated preoperative and postoperative role of NLR in the prognosis of renal cell carcinoma (RCC). Only the data before the intervention was included in the analysis and the results for RFS and CSS were combined as RFS/CSS. Thus, a total of 13 studies involved 2391 patients with urinary cancers evaluating OS and 9 studies including 1923 cases for RFS/CSS were analyzed in our meta-analysis.

As shown in Table 1, ethnicity background of patients was classified as Caucasian and Asian population. The number of patients in each study ranged from 48 to 678. A total of 11 studies explored NLR in the prognosis of RCC and 2 of prostate cancer, 2 of bladder cancer and 2 of urothelial carcinoma, respectively. The cut-off value applied in each study was not consistent ranged from 2 to 5.

**Table 1:** characteristics of all the studies.

Author	Year	Country	Ethnicity	Stage	Type	Number	Cut-off	Survival analysis	Follow-up (months) (median or/and range)
**Yoshio Ohno**	2010	Japan	Asian	I–IV	RCC	192	2.7	RFS	6–232
**Daniel Keizman**	2012	USA	Caucasian	NR	RCC	109	3	OS	37(5–85)
**Yoshio Ohno**	2012	Japan	Asian	I–III	RCC	250	2.7	RFS	21–129
**Bulent Cetin**	2013	Turkey	Caucasian	NR	RCC	100	3.04	OS	15(1–53)
**Yoshio Ohno**	2013	Japan	Asian	I–IV	RCC	48	4	OS	11.7(1–114)
**M Pichler**	2013	Austria	Caucasian	I–IV	RCC	678	3.3	OS,CSS,	0–130
**Minoru Kobayashi**	2013	Japan	Asian	NR	RCC	58	3.32	OS	12(1.1–48.9)
**P Fox**	2013	Austria	Caucasian	NR	RCC	362	2	OS	NR
**Patrice Forget**	2013	Belgium	Caucasian	NR	RCC	227	5	OS,RFS	NR
**M Santoni**	2013	Italy	Caucasian	NR	RCC	97	3	OS	46.9(39.9–53.9)
**Shingo Hatakeyama**	2013	Japan	Asian	III–IV	RCC	85	NR	OS	26
**K Shafique**	2012	UK	Caucasian	NR	Prostate	265	5	OS	30
**Tatsuo Gondo**	2012	Japan	Asian	I–IV	Bladder	189	2.5	CSS	25.1(2.1–127.9)
**L. Spencer Krane**	2013	USA	Caucasian	NR	Bladder	68	2.5	OS,CSS	NR
**Takeshi Azuma**	2013	Japan	Asian	I–IV	Urothelial carcinoma	137	2.5	RFS,CSS	60.9(1.9–187.3)
**Anthony Linton**	2013	Austria	Caucasian	I–IV	Prostate	112	5	OS	NR
**Orietta Dalpiaz**	2013	Austria	Caucasian	I–IV	Urothelial carcinoma	182	2.7	OS,CSS	0–199

RCC: renal cell carcinoma; OS: overall survival; RFS: recurrence-free survival; CSS: cancer-specific survival; NR: not reported; Cut-off: cut-off value of neutrophil-to-lymphocyte ratio (NLR) applied in each study.

### Outcome from eligible studies

**Figure 2:**
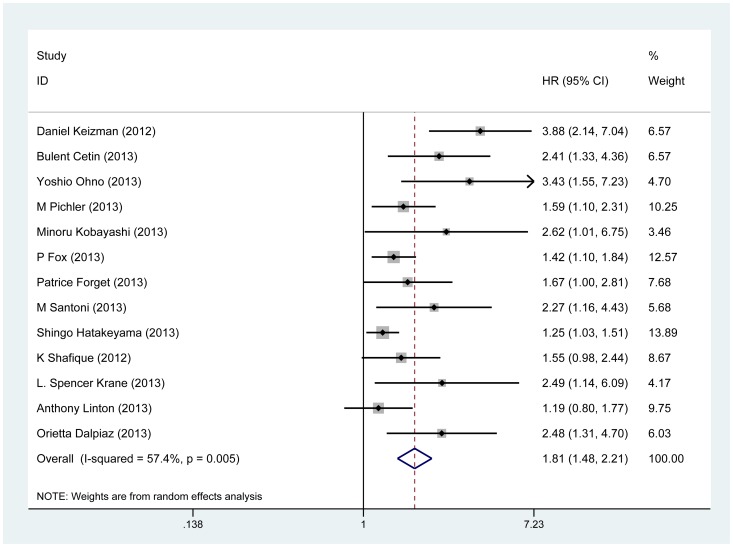
Forrest plots of studies evaluating hazard ratios (HRs) of NLR for overall survival.

As was shown in Table 2, we found elevated NLR predicted a worse outcome with the pooled HR of 1.81 (95%CI: 1.48–2.21; P_heterogeneity_ = 0.005) for 13 studies evaluating OS (Figure 2). Similarly predictive role of NLR for RFS/CSS was also investigated with combined HR of 2.07 (95% CI: 1.65–2.6; P_heterogeneity_ = 0.849).

**Table 2:** Main results.

Outcome	Variable	Number of studies	Model	HR(95% CI)	P_heterogeneity_
**OS**	All	13	Random	1.81(1.48,2.21)	0.005
	Cancer type				
	RCC	9	Random	1.9(1.47,2.45)	0.003
	Prostate	2	Fixed	1.33(0.99,1.8)	0.392
	Ethnicity				
	Caucasian	10	Random	1.81(1.47,2.24)	0.052
	Asian	3	Random	2.05(0.99,4.25)	0.017
	Sample size				
	Large	8	Random	1.76(1.39,2.22)	0.035
	Small	5	Random	2.1(1.3,3.39)	0.018
**RFS/CSS**	All	9	Fixed	2.07(1.65,2.6)	0.849
	Cancer type				
	RCC	4	Fixed	1.83(1.35,2.48)	0.709
	Bladder	2	Fixed	2.2(1.27,3.8)	0.447
	Urothelial	3	Fixed	2.58(1.66,4.01)	0.784
	Ethnicity				
	Caucasian	4	Fixed	1.86(1.33,2.61)	0.471
	Asian	5	Fixed	2.26(1.66,3.09)	0.929

RCC: renal cell carcinoma

Subgroup analyses by cancer type showed that high NLR yielded a worse OS in RCC (HR = 1.9, 95%CI: 1.47–2.45; P_heterogeneity_ = 0.003) and a poor RFS/CSS in RCC (HR = 1.83, 95%CI: 1.35–2.48; P_heterogeneity_ = 0.709), bladder cancer (HR = 2.2, 95%CI: 1.27–3.8; P_heterogeneity_ = 0.447) and urothelial carcinoma (HR = 2.58, 95%CI: 1.66–4.01; P_heterogeneity_ = 0.784).

In the subgroup analyses by ethnicity, we found that no matter the patients were Asian or Caucasian, elevated NLR was still a poor predictor for RFS/CSS (Caucasian: HR = 1.86, 95%CI: 1.33–2.61; P_heterogeneity_ = 0.471; Asian: HR = 2.26, 95%CI: 1.66–3.09; P_heterogeneity_ = 0.929) but only showed similar role for OS in Caucasian population (Caucasian: HR = 1.81, 95%CI: 1.47–2.24; P_heterogeneity_ = 0.052; Asian: HR = 2.05, 95%CI: 0.99–4.25; P_heterogeneity_ = 0.017).

Further analyses of studies evaluating OS by sample size (studies with more than 100 cases were classified as “large”, and studies with less than 100 cases were classified as “small”) also revealed that high NLR remained to be a worse prognostic marker regardless of sample size (large: HR = 1.76, 95%CI: 1.39–2.22; P_heterogeneity_ = 0.035; small: HR = 2.1, 95%CI: 1.3–3.39; P_heterogeneity_ = 0.018).

### Heterogeneity

To explore the potential source of heterogeneity among studies for OS, meta-regression was conducted by using variables as year of publication, ethnicity, cancer type and sample size (≥100 vs. <100). The results showed that year of publication (p = 0.365), ethnicity (p = 0.96), cancer type (p = 0.797) and sample size (p = 0.671) did not contribute to the source of heterogeneity

### Publication bias

**Figure 3:**
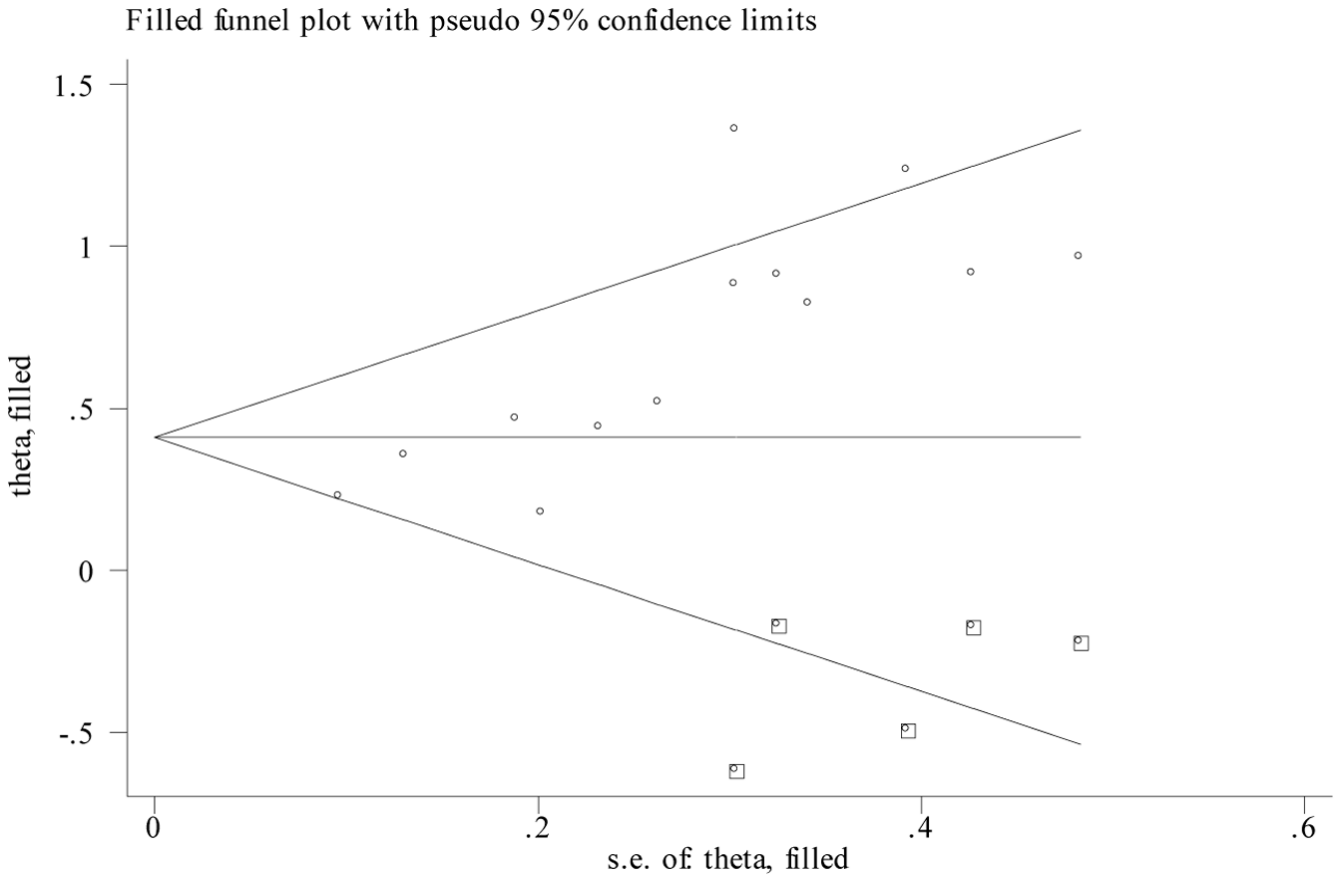
Funnel plot adjusted with trim and fill method for overall survival. Circles: included studies. Diamonds: presumed missing studies.

Publication bias was evaluated using Begg's funnel plot and the Egger's linear regression test. However, publication bias was detected for OS (P = 0.024 for Begg's test and P<0.001 for Egger's test) and RFS/CSS (P = 0.016 for Begg's test and P = 0.005 for Egger's test). Thus, a trim and fill method was performed and pooled HRs were recalculated with hypothetically non-published studies to assess the asymmetry in the funnel plot. The recalculated results did not change significantly for OS (HR = 1.51, 95%CI: 1.22–1.86; P_heterogeneity_<0.001; Figure3) and RFS/CSS (HR = 1.95, 95%CI: 1.57–2.42; P_heterogeneity_ = 0.78), indicating the stability of the results.

## Discussion

This meta-analysis including 17 studies involving 3159 cases with urinary cancers showed that elevated NLR indeed predicted a worse clinical outcome. Subgroup analyses revealed that poor OS with high NLR could be found in RCC and worse RFS/CSS in RCC, bladder cancer and urothelial carcinoma. Elevated NLR was a significant prognostic marker for worse RFS/CSS regardless of ethnicity background and predicted poor OS in Caucasian population but not in Asian patients. When analyzed by sample size, similarly significant results were found in both large and small sample studies. Meta-regression was utilized to investigate the source of heterogeneity. However, none of the variables listed above contributed to the heterogeneity. As publication bias was observed, a trim and fill method was conducted to recalculate the adjusted HRs and we did not find different results for OS and RFS/CSS, suggesting the stability of the analysis.

Increasing evidence showed the association of inflammation and cancer [30] and was helpful in the prevention and treatment of cancer, such as the anti-inflammation therapy of bladder cancer [31], [32]. An enhanced neutrophil response and/or suppression of lymphocyte leading to a high NLR might promote carcinogenesis and inhibit antitumor immune response [33], [34]. Molecular signaling and pathway triggered by inflammatory mediators could promote cancer cell proliferation angiogenesis and metastasis, thus impacting the tumor response to therapies [35]. Additionally, some studies showed that elevated NLR indicate an increased risk of ischemic cardiovascular diseases [36], high mortality in patients with bacteraemia [37] and raised gastrointestinal morbidity and mortality [38], [39] which may underlie the poor prognosis of patients with an increased NLR. Nowadays, tumor stage and other clinical parameters such as PSA and Gleason grade were applied to obtain prognostic information and be helpful in choice of appropriate treatment strategies for patients with urinary cancers. Peripheral blood tests before treatment or at the time of diagnosis may reflect inflammatory conditions within the tumor. NLR calculated from the convenient and cheap test could provide appropriate prognostic information for the patients in the treatment of urinary cancers.

Several limitations of our study should be considered. First, the studies retrieved in the analysis were full text in English searched on PubMed which might be responsible for the observed publication bias though not affecting the results by trim and fill method. Second, marked heterogeneity of studies were found in OS group. And no variables analyzed in the meta-regression contributed to the heterogeneity. In fact, the presence of heterogeneity may result from many other factors, including age distribution, gender, lifestyle and so on. Due to lack of detailed data, we could not use these variables in the meta-regression. Third, the number of studies and participants for analysis was not large enough for further subgroup analysis such as only one study investigated NLR for OS in bladder cancer and urothelial carcinoma, respectively. And only 3 studies on Asian population yielded a trend of poorly predictive role of NLR for OS. Fourth, due to lack of data, the association between NLR and other clinical parameters such as Prostate-specific antigen (PSA) was not explored. Thus, additional well-designed studies with more cancer types and larger sample size are needed to present more reliable results.

In conclusion, the evidence from the meta-analysis of published studies showed that elevated NLR was a poor predictor for survival in patients with urinary cancers. However, attention should be paid due to the limitations listed above. To better understand the role of NLR in the prognosis of urinary cancers and apply the simple and cheap prognostic factor in clinical, more large-scale and standard investigations should be conducted.

## Supporting Information

Checklist S1 **PRISMA 2009 Checklist.**
(DOC)
